# OsWRKY62 and OsWRKY76 Interact with Importin α1s for Negative Regulation of Defensive Responses in Rice Nucleus

**DOI:** 10.1186/s12284-022-00558-4

**Published:** 2022-02-20

**Authors:** Xiaohui Xu, Han Wang, Jiqin Liu, Shuying Han, Miaomiao Lin, Zejian Guo, Xujun Chen

**Affiliations:** 1 Key Laboratory of Pest Monitoring and Green Management, MOA, Joint Laboratory for International Cooperation in Crop Molecular Breeding; Department of Plant Pathology, China Agricultural University, Beijing, 100193 China; 2 College of Modern Science and Technology, China Jiliang University, Hangzhou, 310018 China

**Keywords:** AvrPib, Importin α, Nuclear localization signal, WRKY, *Magnaporthe oryzae*, *Oryza sativa*

## Abstract

*Background* OsWRKY62 and OsWRKY76, two close members of WRKY transcription factors, function together as transcriptional repressors. OsWRKY62 is predominantly localized in the cytosol. What are the regulatory factors for OsWRKY62 nuclear translocation? *Results* In this study, we characterized the interaction of OsWRKY62 and OsWRKY76 with rice importin, OsIMα1a and OsIMα1b, for nuclear translocation. Chimeric OsWRKY62.1-GFP, which is predominantly localized in the cytoplasm, was translocated to the nucleus of *Nicotiana benthamiana* leaf cells in the presence of OsIMα1a or OsIMαΔIBB1a lacking the auto-inhibitory importin β-binding domain. OsIMαΔIBB1a interacted with the WRKY domain of OsWRKY62.1, which has specific bipartite positively charged concatenated amino acids functioning as a nuclear localization signal (NLS). Similarly, we found that OsIMαΔIBB1a interacted with the AvrPib effector of rice blast fungus *Magnaporthe oryzae*, which contains a scattered distribution of positively charged amino acids. Furthermore, we identified a nuclear export signal (NES) in OsWRKY62.1 that inhibited nuclear transportation. Overexpression of *OsIMα1a* or *OsIMα1b* enhanced resistance to *M. oryzae*, whereas knockout mutants decreased resistance to the pathogen. However, overexpressing both *OsIM*α*1a* and *OsWRKY62.1* were slightly more susceptible to *M. oryzae* than *OsWRKY62.1* alone. Ectopic overexpression of *OsWRKY62.1*-*NES* fused gene compromised the enhanced susceptibility of OsWRKY62.1 to *M. oryzae*. *Conclusion* These results revealed the existence of NLS and NES in OsWRKY62. OsWRKY62, OsWRKY76, and AvrPib effector translocate to nucleus in association with importin α1s through new types of nuclear localization signals for negatively regulating defense responses.

**Supplementary Information:**

The online version contains supplementary material available at 10.1186/s12284-022-00558-4.

## Introduction

In eukaryotic cells, the nuclear envelope governs the nuclear and cytoplasmic trafficking pathways and provides an important feature to control the specificity and spatio-temporal signaling events. Translocation proteins are generally recognized by the nuclear transport receptors (NTRs) based on the nuclear localization signals (NLSs) or nuclear export signals (NESs) on the cargo proteins (Krebs et al. 2010). Importin α, an adaptor of NTRs, consists of tandem armadillo repeats and an auto-inhibitory importin β-binding (IBB) domain, which can bind to the NLS of a cargo and importin β (IMβ), respectively (Cook et al. 2007; Kobe 1999). Then, the ternary importin α/β-cargo complex passes through the nuclear pore complexes (NPCs) through transient interactions between importin β and Phe/Gly-repeat nucleoporins (Nups) of the NPCs that create a selective permeability barrier (Schmidt and Görlich 2016). Conversely, chromosome maintenance protein1 (CRM1/XPO1, or exportin 1), a nuclear export receptor, binds to the cargo proteins with NESs and to RanGTP inside the nucleus, traverses the NPCs, and releases cargo into the cytoplasm (Haasen et al. 1999; Monecke et al. 2014; Ossareh-Nazari et al. 2001). Such dynamic changes of nucleocytoplasmic proteins have been implicated in the regulation of plant developmental and environmental responses (Yang et al. 2017).

Genetic screens for suppressors of the autoimmune mutant, *snc1* (*suppressor of npr1-1, constitutive1*), have led to the identification of *mos* (*modifiers of snc1*) mutants (Zhang and Li 2005). Among the *MOS* genes, *MOS3* and *MOS7* encode Nup96 and Nup88 of the NPCs, respectively. MOS7 is required for basal resistance, effector-triggered immunity (ETI), and resistance against biotrophic, hemibiotrophic, and necrotrophic pathogens (Cheng et al. 2009; Genenncher et al. 2016). MOS7 is verified to promote the nuclear accumulation of the immunoregulatory proteins such as EDS1 (enhanced disease susceptibility 1), NPR1 (nonexpresser of pathogenesis-related gene 1), and MPK3 (mitogen-activated protein kinase 3). It has been demonstrated that the balance of EDS1 in the cytosol and nucleus is required for efficient basal immune and toll/interleukin1 receptor domain-containing nucleotide-binding leucine-rich repeat (TNL) R protein-triggered resistance (García et al. 2010). Sufficient abundance of MPK3 protein in the nucleus is important for full immunity to *Botrytis cinerea* in Arabidopsis (Genenncher et al. 2016). Moreover, CPR5 (constitutive expresser of *PR* gene 5) is a novel transmembrane nucleoporin, which associates with NPC core scaffold to allow massive nuclear influx of diverse stress-related signaling cargos after activation of immunoreceptors (Gu et al. 2016). The data clearly indicate an important role of nucleocytoplasmic trafficking, especially nuclear import protein, in plant innate immunity.

Nuclear cargo proteins usually consist of importin α-binding NLSs, which are monopartite or bipartite (separated by 10–12 residues at the linker region) sequence motifs enriched in basic amino acids of three to five residues (Chang et al. 2012). In *Arabidopsis*, *MOS6*, which encodes importin α3 from the nine *Arabidopsis* importin α isoforms, is required for TNL *R* gene *snc1*-mediated resistance against a virulent oomycete pathogen (Palma et al. 2005) and demonstrated to be the main NTR of SNC1 (Lüdke et al. 2021), importing proteins involved in defense signaling in the nucleus. Moreover, nuclear translocation of Tartary buckwheat FtMYB16 is mediated by importin α1 to repress rutin biosynthesis (Li et al. 2019). Furthermore, plant pathogens may take advantage of the plant protein transport system to deliver virulent effector proteins into the host cytoplasm or periplasmic space to interfere with and manipulate host functions. Rice importin α1a and *Arabidopsis* importin αs interact with the bipartite NLSs of *Agrobacterium tumefaciens* virulence proteins VirD2 and VirE2, respectively (Bhattacharjee et al. 2008; Chang et al. 2014). Recently, rice importin α1a and importin α1b have shown to be necessary for nuclear import transcription activator-like effectors (TALEs), which are the secreted virulence proteins of *Xanthomonas oryzae* pv. *oryzae* (*Xoo*) and *X. oryzae* pv. *oryzicola* (*Xoc*), the causal agents of bacterial leaf blight and leaf streak, respectively (Hui et al. 2019). Nuclear localization of TALEs increases host susceptibility and modulates host gene expression (Hui et al. 2019; Szurek et al. 2001). Conversely, nuclear localization of effector AvrPib from rice blast fungus *Magnaporthe oryzae* is required for its avirulence function (Zhang et al. 2018). The results suggest that nuclear transportation of proteins play a significant role in response to biotic and abiotic challenges.

OsWRKY62 and OsWRKY76, belonging IIa subgroup of WRKY transcription factors (TFs), function negatively in disease resistance against *M. oryzae* and *Xoo* (Liu et al. 2016; Peng et al. 2008; Wu et al. 2005; Yokotani et al. 2013). However, OsWRKY62 may act as a positive regulator of defense when it forms heterocomplex with a strong transactivator OsWRKY45 (Fukushima et al. 2016). Stable and transient expression of OsWRKY62.1-GFP (an alternative splicing product of OsWRKY62) shows cytosolic localization in unknown structures or as aggregates (Liu et al. 2016). Interestingly, OsWRKY62.1 is localized in the nuclei when it interacts with OsWRKY76.1 (an alternative splicing product of OsWRKY76) or XA21, a rice pattern recognition receptor for *Xoo* (Liu et al. 2016; Park and Ronald 2012), implying a conditioned nuclear import of OsWRKY62.1 for its regulatory function. In a yeast two-hybrid (Y2H) cDNA library screening, we found that OsWRKY76.1 interacted with rice importin α1a (OsIMα1a). Analysis indicated that OsWRKY62.1 also interacted with OsIMα1a and its close homolog OsIMα1b through a new type NLS sequence. Furthermore, dissection of OsWRKY62.1 sequence revealed the existence of a NES sequence, which played a determinant role for OsWRKY62.1 localization. This study provided the relationship of OsWRKY62 localization and disease resistance and widened the knowledge about NLSs.

## Results

### OsIMα1a and OsIMα1b Interact with OsWRKY62 and OsWRKY76 Proteins

**Fig. 1:**
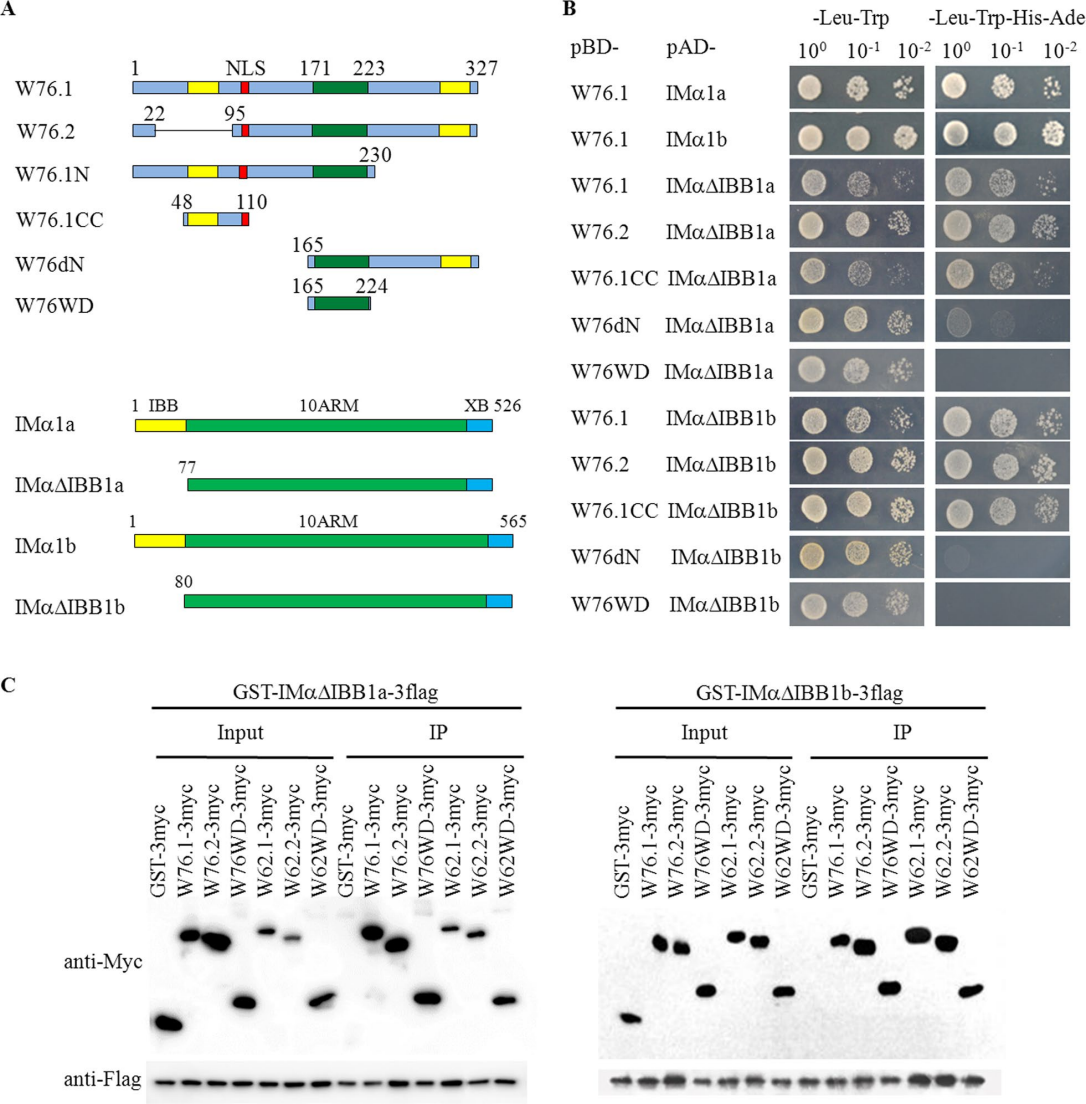
Interaction of OsIMα1a and OsIMα1b with OsWRKY76. **a** Schematic diagrams of OsWRKY76.1 (W76.1), OsIMα1a (IMα1a), OsIMα1b (IMα1b), and their deletion mutants. NLS, nuclear localization signal; WD, WRKY domain; ARM, armadillo repeats; IBB, importin-β-binding domain; XB, exportin binding domain. **b** W76.1 and its deletion mutants were fused to the Gal4 DNA-binding domain (BD). IMα1a, IMα1b, IMαΔIBB1a, and IMαΔIBB1b were fused to the Gal4 activation domain (AD). Yeast cells with serial dilutions (10^0^, 10^–^^1^, and 10^–^^2^) were incubated in synthetic dropout medium lacking Leu and Trp (left panel) or Leu, Trp, His, and Ade (right panel) and photographed 3 days after plating. **c** Pull-down assays of OsIMαΔIBB1a and OsIMαΔIBB1b interacting with OsWRKY62 and OsWRKY76. All proteins were purified with their N-termini fused with GST and their C-termini fused with 3×flag or 3×myc tag. Each protein (about 1 µg) with its corresponding tag combination was incubated at 4 °C for 3 h in the immunoprecipitation (IP) buffer. The protein complexes were precipitated with anti-Flag affinity gel, washed five times with the IP buffer, and separated on 10% SDS-PAGE gels. The proteins were detected by western blots with anti-Myc and anti-Flag antibodies. Similar results were obtained from three repeats. NLS, nuclear localization signal; WD, WRKY domain

OsIMα1a was one of the interacting candidates of OsWRKY76 in a yeast two-hybrid (Y2H) screening of cDNA library. To confirm the interaction of OsWRKY76.1 with OsIMα1a in yeast cells, OsWRKY76.1 and its deletion mutants were fused with the Gal4 DNA-binding domain (in the bait vector pBD) and the coding range of OsIMα1a without the auto-inhibitory IBB domain (IMαΔIBB1a) was fused with the activation domain (in the prey vector pAD) (Fig. 1a). The bait and prey plasmid combinations were transformed into yeast cells. Interaction was observed between AD-IMαΔIBB1a and BD-W76.1, BD-W76.2, or BD-W76.1CC containing a potential coiled-coil (CC) domain with the predicted NLS of KKRSR at the C-terminus, indicated by yeast growth on the selective media lacking Leu, Trp, His, and Ade (Fig. 1b; Additional file 1: Figure S1A). Deletion of the N-terminal OsWRKY76.1 (W76dN) almost abolished its interaction with OsIMα1a. Similarly, we examined rice importin α1b (OsIMα1b), a close homolog of OsIMα1a (Additional file 1: Figure S2), and found that its IBB deletion mutant (OsIMαΔIBB1b) interacted similar to how OsIMαΔIBB1a did with OsWRKY76.1 and its deletion mutants. Further, we examined interactions of OsWRKY62.1, a paralog of OsWRKY76.1 (Wu et al. 2005), with the two importin α1s. Since BD-W62.1, -IMαΔIBB1a, and -IMαΔIBB1b exhibited weak autoactivation (Additional file 1: Figure S1; Liu et al. 2016), we tested on the N-terminal deletion mutant (pBD-W62dN) and found that W62dN could not interact with IMαΔIBB1a or IMαΔIBB1b in yeast cells (Additional file 1: Figure S1C).

Protein pull-down assays were performed to analyze the interaction between the two WRKY TFs and importin α1s in vitro. OsWRKY62.1 and OsWRKY76.1 were sandwiched between glutathione *S*-transferase (GST-) and -3 × myc tags at their N-termini and C-termini, respectively, whereas OsIMα1a or OsIMα1b were constructed between GST- and -3 × flag tags. The mixture of recombinant proteins was pulled down with anti-Flag affinity gel. As shown in Fig. 1c, GST-IMαΔIBB1a-3flag and GST-IMαΔIBB1b-3flag formed complexes with both OsWRKY62.1 and OsWRKY76.1 under the assay conditions. GST-IMαΔIBB1a-3flag and GST-IMαΔIBB1b-3flag were also found to interact with OsWRKY62.2 (W62.2-3myc) and OsWRKY76.2 (W76.2-3myc), encoding the alternative spliced proteins (Liu et al. 2016), and the WRKY domains (WDs) W62WD-3myc and W76WD-3myc, respectively. Thus, the results revealed that the interactions of IMαΔIBB1a and -1b with OsWRKY62 were different between the in vitro pull-down analysis and in yeast cells.

### Interaction of OsIMα1a and OsIMα1b with OsWRKY62 and OsWRKY76 was Primarily in the Nucleus

**Fig. 2:**
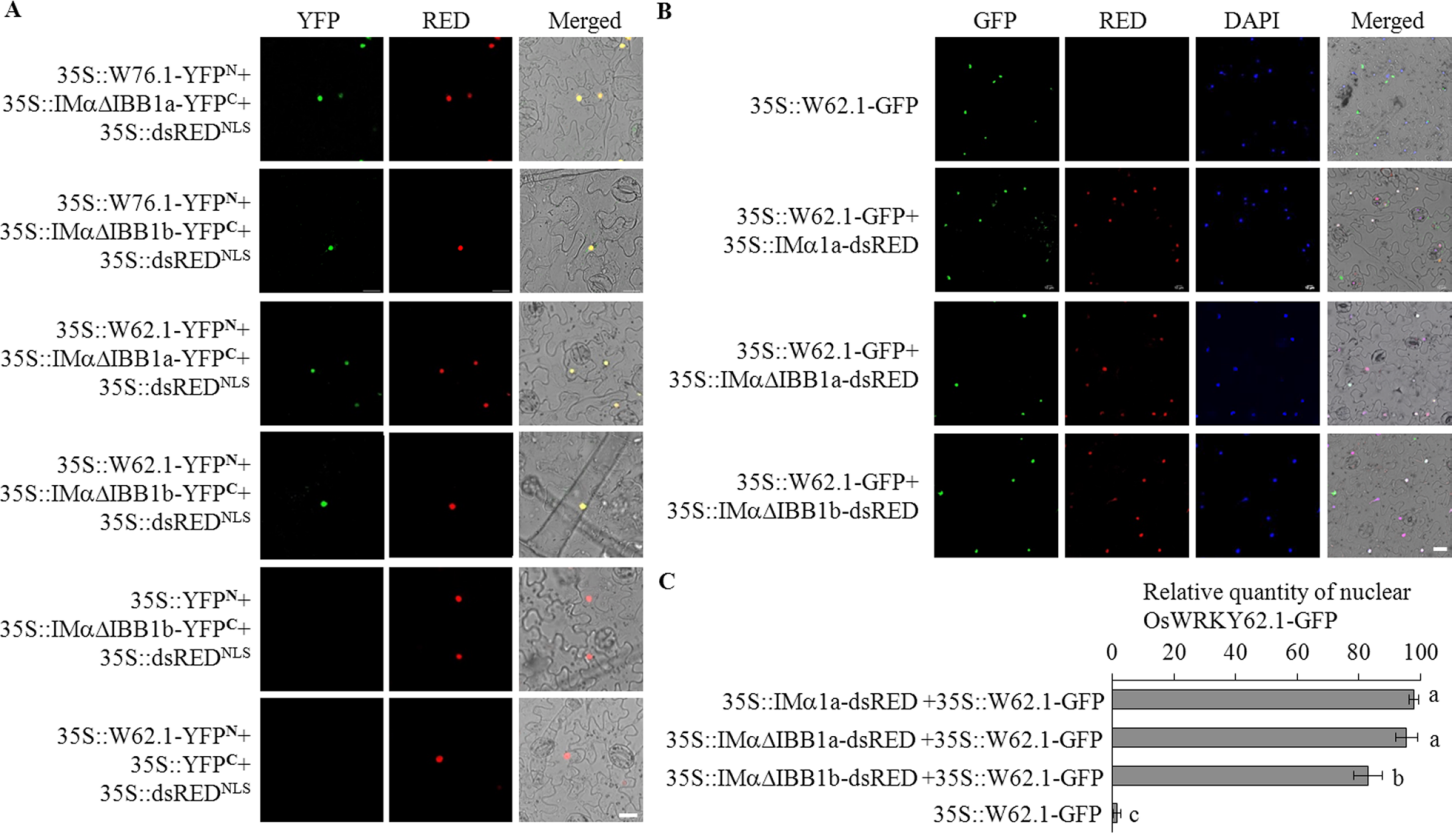
OsIMα1a and OsIMα1b interacting with OsWRKY62.1 and OsWRKY76.1 primarily in the nuclei. **a** BiFC visualizations of OsWRKY62.1 (W62.1) and OsWRKY76.1 (W76.1) interacting with OsIMα1a and OsIMα1b. W62.1 and W76.1 were fused in frame with YFP N-terminal region (YFP^N^) and IMαIBB1a and IMαIBB1b were fused with YFP C-terminal region (YFP^C^). The plasmids indicated were introduced into *N. benthamiana* leaves through agroinfiltration method. Confocal images were taken 72 h after the treatments. Red fluorescence (dsRED^NLS^) shows nuclear localization. From left panels to right: YFP images (YFP), dsRED images (RED), and combined YFP and RED in the bright field (Merged). **b** Co-localization of OsW62.1 with OsIMα1a and OsIMα1b. Plasmid 35S::W62.1-GFP or in combination with plasmid indicated was delivered into *N. benthamiana* leaves through agroinfiltration. Images were photographed 72 h after the treatments. DAPI (4',6-diamidino-2-phenylindole) for nuclear staining. From left panels to right: GFP, RED, DAPI, and the bright field image combined the fluorescent images (Merged). **c** Relative quantity of OsWRKY62.1-GFP localized in the nuclei. More than 100 cells from (**b**) were counted. Significant differences (Duncan's multiple range test; α = 0.05) compared with 35S::W62.1-GFP only are listed in the figure. Bar = 20 µm

To validate the interactions of OsIMα1a and OsIMα1b with OsWRKY76.1 and OsWRKY62.1 *in planta*, their proteins were fused in frame with the N- or C-terminal region of the yellow fluorescent protein (YFP) and controlled by the cauliflower mosaic virus 35S promoter for the bi-molecular fluorescence complementation (BiFC) assay. YFP fluorescence was detected in the epidermal cells of *Nicotiana benthamiana* leaves transformed with 35S::IMαΔIBB1a-YFP^C^ and 35S::W76.1-YFP^N^ or 35S::W62.1-YFP^N^ plasmid, and overlapped with the red fluorescence of dsRED^NLS^ (dsRED with additional NLS sequence), indicating the interaction at the nuclei (Fig. 2a). OsIMαΔIBB1b-YFP^C^ also interacted with YFP^N^ fused OsWRKY76.1 and OsWRKY62.1 in the nuclei.

OsWRKY62.1 is detected exclusively in unknown structures or as aggregates in rice plants stably transformed with 35S::W62.1-GFP and in transiently expressed *N. benthamiana* leaves (Liu et al. 2016). Interaction of OsIMαΔIBB1a with OsWRKY62.1 in the nuclei encouraged us to examine OsWRKY62.1 redistribution in plant cells. Plasmids of 35S::IMα1a-dsRED and 35S::W62.1-GFP were introduced into *N. benthamiana* leaves via *Agrobacterium*-mediated infiltration. Co-expression of 35S::W62.1-GFP with 35S::IMα1a-dsRED brought a higher percentage of OsWRKY62.1-GFP into the nuclei than 35S::W62.1-GFP did alone (Fig. 2b, c). Similar nuclear localization of OsWRKY62.1-GFP was observed by co-infiltration of 35S::W62.1-GFP with 35S::IMαΔIBB1a-dsRED or 35S::IMαΔIBB1b-dsRED. The IBB deletion mutants of IMα1s interacting with OsWRKY62.1 in the nuclei promoted us to investigate whether the mutants affects association with IMβ1. Both IMα1a-YFP^C^ and IMαΔIBB1a-YFP^C^ interacted with IMβ1-YFP^N^ at the similar extent in the BiFC assays (Additional file 1: Figure S3A). Furthermore, the increase in nuclear localization of OsWRKY62.1 fusion proteins was confirmed by analysis of 35S::W62.1-GFP and CDU::IMα1a (a maize *ubiquitin* gene promoter controlled OsIMα1a) crossed progeny (35S::W62.1-GFP/CDU::IMα1a; Additional file 1: Figure S3B). The results suggested that OsWRKY62.1 interacts with OsIMα1a and OsIMα1b for nuclear translocation.

### OsIMα1a Interaction with the Concatenated Basic Amino Acids in the WRKY Domain of OsWRKY62 and AvrPib

**Fig. 3:**
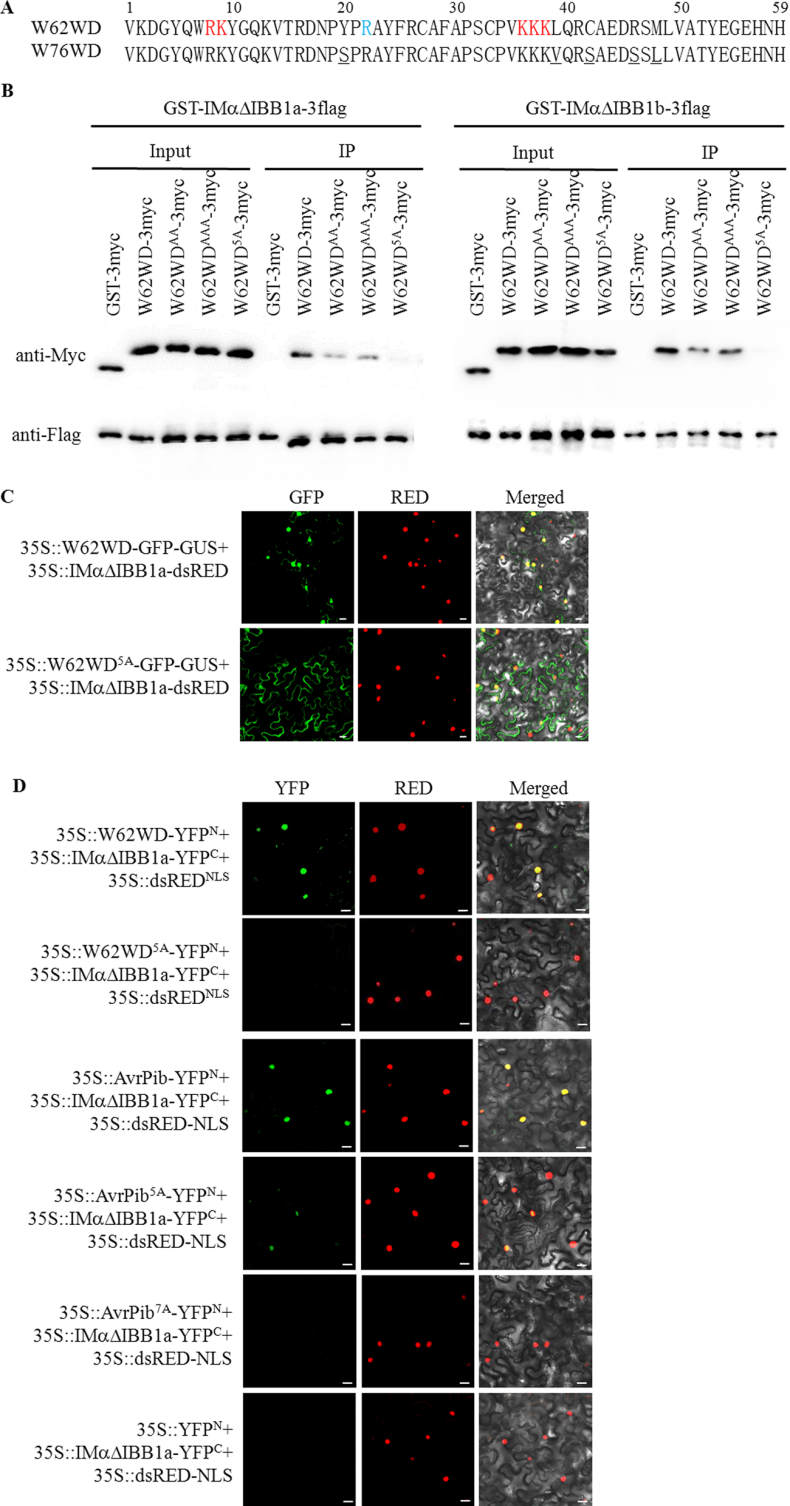
Interaction of OsIMαΔIBB1a and OsIMαΔIBB1b with the WD of OsWRKY62. **a** WD sequences of OsWRKY62 and OsWRKY76. The basic amino acids in red are mutated to Alanine. The amino acid differences between W62WD and W76WD are underlined in W76WD. **b** Pull-down assays of OsIMαΔIBB1a and OsIMαΔIBB1b interacting with W62WD and its mutants. The W62WD and its mutants were sandwiched with GST and 3myc tags and expressed. Each protein (about 1 µg) was incubated with GST-IMαΔIBB1a-3flag or GST-IMαΔIBB1b-3flag at 4 °C for 3 h in the IP buffer. The protein complexes were precipitated, washed five times with the IP buffer, separated on 10% SDS-PAGE gels, and detected by western blots with anti-Myc and anti-Flag antibodies. GST-3myc was used as a negative control. Similar results were obtained from three repeats. WD^AA^, WD^AAA^, and WD^5A^ for RK, KKK, and both of them in W62WD, respectively, were all mutated to Alanines. **c** The concatenated basic amino acids in W62WD were important for nuclear localization. *W62WD* and *W62WD*^*5A*^ were cloned in frame with *GFP-GUS* chimeric gene, respectively. The generated plasmid in combination with *35S::IMαΔIBB1a-dsRED* were introduced into the leaf cells of *N. benthamiana*. Confocal images were taken 72 h after the agroinfiltration. From left panel to right: GFP images (GFP), dsRED images (RED), and combined GFP and RED in the bright field (Merged). **d** Interactions of IMαΔIBB1a with W62WD and AvrPib. W62WD, AvrPib, and their mutants were fused in frame with YFP N-terminal region (YFP^N^), and IMαΔIBB1a was fused with YFP C-terminal region (YFP^C^). The plasmids indicated were transformed into *N. benthamiana* leaves. Confocal images were taken at 72 h after the treatments. Red fluorescence (35S::dsRED^NLS^) shows nuclear localization. From left panels to right: YFP images (YFP), dsRED images (RED), and combined YFP and RED in the bright field (Merged). Bar = 20 µm

Importin αs have been demonstrated to interact with NLSs (Chang et al. 2012). The interactions of OsWRKY62 and OsWRKY76 WDs with OsIMαΔIBB1a-3flag and OsIMαΔIBB1b-3flag implied the potential existence of NLS-like sequence in the WDs (Fig. 2c). The WD of OsWRKY62 (W62WD) comprised of ^8^RK and ^36^KKK sequences 26 amino acid residues apart from each other (Fig. 3a). Mutation in one of the two sites significantly decreased its affinity to OsIMαΔIBB1a-3flag and OsIMαΔIBB1b-3flag, and the interactions were almost completely inhibited when the basic amino acids of both loci were substituted by A residues (W62WD^5A^, Fig. 3b). Furthermore, the W62WD was fused in frame with the chimeric GFP-GUS protein to prevent passive diffusion of the expressed protein into the nuclear compartment. The GFP fluorescence signal of 35S::W62WD-GFP-GUS was mainly in the nuclei of *N. benthamiana* leaf cells, while the fluorescence of 35S::W62WD^5A^-GFP-GUS was mostly in the cytoplasm (Fig. 3c). Therefore, from the BiFC assays, the W62WD, but not the mutated W62WD^5A^, was found to interact with OsIMαΔIBB1a in the nuclei (Fig. 3d). These results indicated that OsWRKY62 contains a specific NLS required for interaction with importin α1a.

Effector AvrPib of *M. oryzae* is secreted into the nucleus of host cells (Zhang et al. 2018). The mature AvrPib is a 52-residue peptide without a typical NLS (Zhang et al. 2015; 2018), but has a scattered distribution of positive-charged amino acids (^29^KK-K^41^-R^45^-R^50^-K^52^-K^70^; Additional file 1: Figure S4A). We examined the possible interaction of OsIMαΔIBB1a with AvrPib using the BiFC assay and found that OsIMαΔIBB1a and AvrPib interacted in the nuclei of *N. benthamiana* leaf cells (Fig. 3d). Fluorescence signals were much less in the combination of the mutated AvrPib^5A^ (underlined K and R mutated to A residues) and OsIMαΔIBB1a (Fig. 3d). This result agrees with that of a previous study, which revealed that the translocation of AvrPib^5A^ to host nuclei is greatly decreased (Zhang et al. 2018). Further, mutation of the K^41^ and R^45^ to A residues (AvrPib^7A^) completely abolished interaction with OsIMαΔIBB1a, suggesting interaction of OsIMα1a with the positive-charged amino acids of AvrPib. Structurally, the five positive-charged amino acids of AvrPib are at the linker regions of β-sheets and form a positive-charged patch on the surface (Zhang et al. 2018). The K^41^ and R^45^ are at the opposite linker and in the β-sheet, respectively (Additional file 1: Figure S4A). The results suggest that the AvrPib conformation possibly enables the five positive-charged patch and the other two basic amino acids (K^41^ and R^45^) loci to interact with OsIMα1a. On the other hand, the simulated W62WD structure showed that the positive-charged amino acids ^8^RK and ^36^KKK in the W62WD were located at the head regions of 2 and 4 β-sheets (Additional file 1: Figure S4B). The W62WD conformation can afford a relative flexible space distance for the basic stretches to associate with OsIMα1a.

### OsIMα1a and OsIMα1b Positively Regulate Disease Resistance

**Fig. 4:**
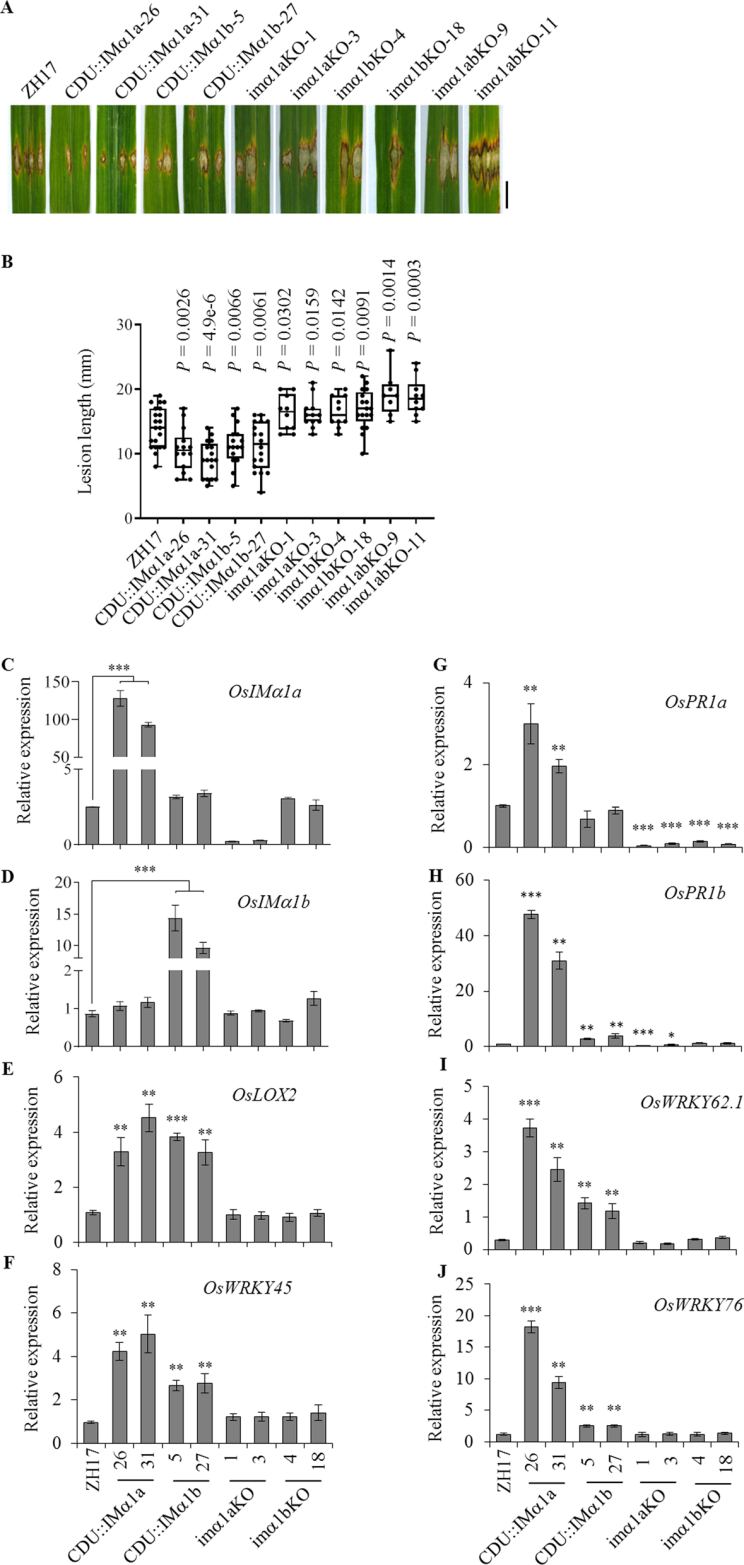
OsIMα1a and OsIMα1b were positively involved in resistance against rice blast pathogen. **a** The transgenic and wild-type (ZH17) plants of about 3-month-old were inoculated with a virulent strain of *M. oryzae* SZ by infiltration. The symptoms were photographed 10 days after the challenge. Bar = 2 cm. **b** Lesion length was measured from the inoculated leaves. *P*-values were calculated by one-tailed Student's *t*-test, n ≥ 8. The experiment was repeated twice with similar results. **c**–**j** Gene expression was determined by qRT-PCR analyses using rice *ubiquitin* gene as an internal standard. Values represent means ± SD (n = 3). Two independent experiments were performed and obtained similar results. CDU for overexpression of *OsIMα1a* and *OsIMα1b*, KO for knockout, and *imα1abKO* for double knockout mutants of *OsIMα1a* and *OsIMα1b*. Significant differences (Student's *t*-test) compared with the wild-type plants are listed in the figure (*P*-value < 0.05, *; < 0.01, **; < 0.001,***)

To investigate biological functions of OsIMα1a and OsIMα1b, we generated their overexpressing (CDU::IMα1a and CDU::IMα1b) and knockout (*imα1aKO* and *imα1bKO*, each gene with a single nucleotide insertion; Additional file 1: Figure S5) plants. Additionally, a double knockout mutant (*imα1abKO*) was obtained by crossing the *imα1aKO* and *imα1bKO* lines. Three-month-old transgenic and control plants were infiltrated with spores of a virulent *M. oryzae* SZ strain. As shown in Fig. 4a, b, the overexpressing plants were more resistant, while the knockout lines were more susceptible to the rice blast fungus as compared to the wild-type ZH17 control. Similar results were obtained on three-week-old plants infected by foliar spray of *M. oryzae* SZ spores (Additional file 1: Figure S6). The data suggested that both *OsIMα1a* and *OsIM*α*1b* are positive regulators of disease resistance against the rice blast pathogen.

Analysis of gene expression revealed that the transferred *OsIMα1a* and *OsIMα1b* genes were highly expressed without significantly affecting each other’s expression (Fig. 4c, d). Lowered expression of *OsIMα1a* in its knockout plants implied a possible feedback regulation of *OsIMα1a*. Furthermore, we analyzed the expressions of defense-related genes. Levels of *OsLOX2* and *OsWRKY45* transcripts were increased in *OsIMα1a* and *OsIMα1b* overexpressing plants (Fig. 4e, f), implying the activation of JA and SA signaling pathways. Interestingly, we noticed a significant decrease of *OsPR1a* levels in *imα1aKO* and *imα1bKO* knockout plants (Fig. 4g), suggesting that *OsIMα1a* and *OsIMα1b* probably participated in the basal immunity. Expression of *OsPR1b* was remarkably elevated in CDU::IMα1b and especially in CDU::IMα1a (Fig. 4h). The pattern of upregulation of *OsWRKY62.1* and *OsWRKY76* was similar to that of *OsPR1b* with higher levels in CDU::IMα1a than CDU::IMα1b (Fig. 4i, j), possibly reflecting the transcription levels of the transgenes. Furthermore, expressions of *OsIMα1a* and *OsIMα1b* genes were induced by PAMPs of flg22 or chitin treatment and only transcriptional level of *OsIMα1a* was increased by *M. oryzae* inoculation at the time point examined (Additional file 1: Figure S7). We also examined the expressions of *OsIMα1a* and *OsIMα1b* genes in *OsWRKY62.1* and *OsWRKY76.1* overexpressing and knockout lines, and found that the transcriptional levels of *OsIMα1a* and *OsIMα1b* were increased in all the analyzed plants (Additional file 1: Figure S7). Collectively, the results suggested that OsIMα1a and OsIMα1b play a positive role in disease resistance and a complicated regulatory mechanism exists among OsIMα1a, OsIMα1b, OsWRKY62, and OsWRKY76.

### Nuclear Localization of OsWRKY62.1 for Its Regulatory Function

**Fig. 5:**
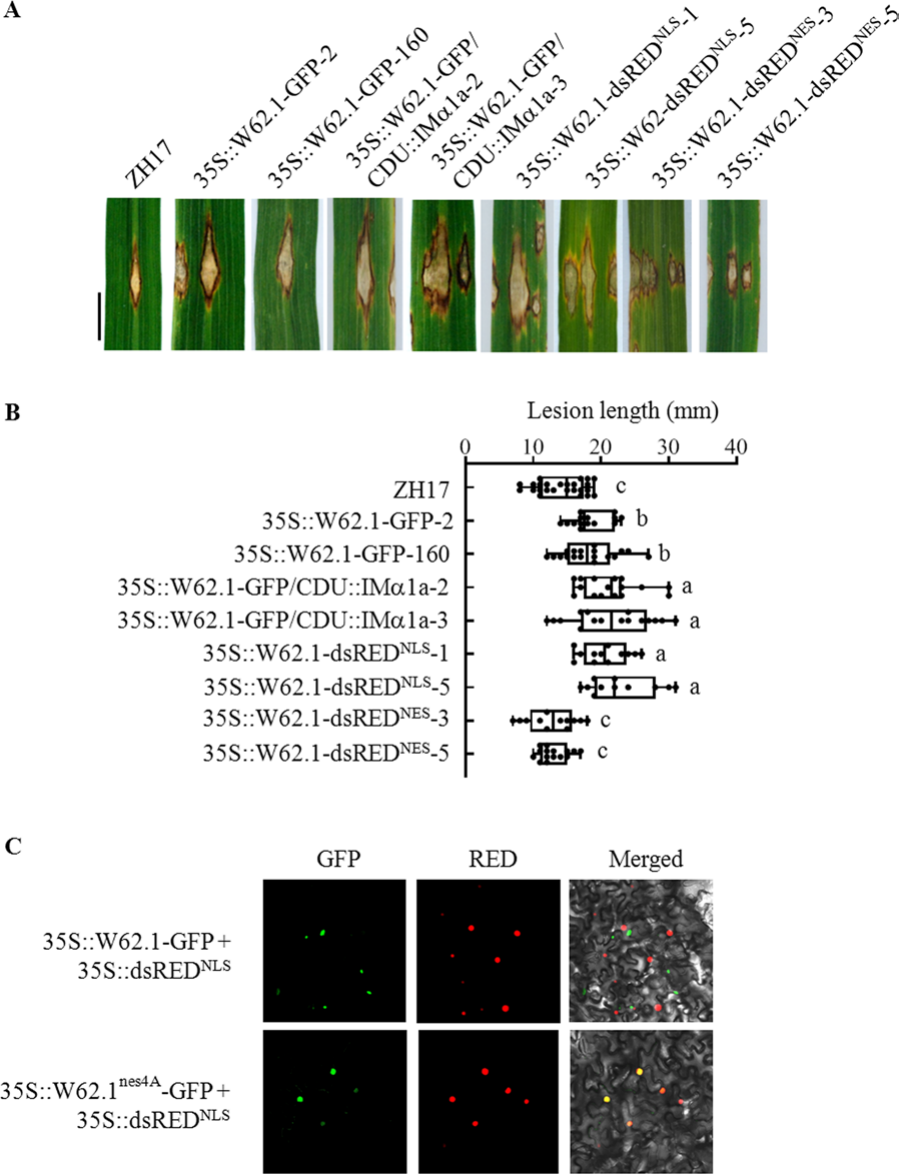
OsIMα1a enhanced OsWRKY62.1 acting as a negative regulator of resistance against rice blast pathogen. **a** The 3-month-old transgenic and wild-type ZH17 plants were inoculated with a virulent strain of *M. oryzae* SZ by infiltration. Symptoms were photographed 10 days after the challenge. Bar = 2 cm. **b** Lesion length was measured from the inoculated leaves. Significant differences (Duncan's multiple range test; α = 0.05) compared with the wild-type plants are listed in the figure. **c** Change of OsWRKY62.1-GFP localization through mutation of NES-like sequence in OsWRKY62.1. The key amino acids in NES-like sequence of OsWRKY62.1 were mutated to A residues (W62.1^nes4A^). The plasmids of 35S::W62.1^nes4A^-GFP and 35S::W62.1-GFP, in combination with 35S::dsRED^NLS^ were introduced into the leaf cells of *N. benthamiana*, respectively. Confocal images were taken 72 h after the agroinfiltration. From left panel to right: GFP images (GFP), dsRED images (RED), and combined YFP and RED in the bright field (Merged). Bar = 20 µm

Since OsIMα1a increased nuclear localization of OsWRKY62.1 (Fig. 2b), we investigated 35S::W62.1-GFP/CDU::IMα1a for resistance against *M. oryzae*. As shown in Fig. 5a, 35S::W62.1-GFP/CDU::IMα1a plants were even more susceptible to *M. oryzae* SZ than its parent 35S::W62.1-GFP and CDU::IMα1a lines, suggesting that OsWRKY62.1 acts downstream of OsIMα1a as a negative regulator of defense against rice blast fungus. Furthermore, we generated 35S::W62.1-dsRED^NLS^ and 35S::W62.1-dsRED^NES^ rice plants, which contained NLS and NES for nuclear and cytosolic localization of the chimeric proteins, respectively. The plants harboring 35S::W62.1-dsRED^NLS^ vector were more susceptible to SZ strain than the ZH17 control, whereas the 35S::W62.1-dsRED^NES^ plants showed no significant change in susceptibility as compared to the ZH17 plants (Fig. 5a). However, the red fluorescence was still not visible in the nuclei of 35S::W62.1-dsRED^NLS^ transgenic rice cells in the normal growth conditions (Additional file 1: Figure S8) The data implied that the NLS sequence was not strong enough to lead the nuclear translocation of W62.1-dsRED^NLS^ protein, even though the dsRED^NLS^ alone was localized in the nucleus (Fig. 2a). OsWRKY62.1 sequence analysis revealed that the ^308^**V**DQ**I**PH**I**P**V**^316^ sequence near the carboxyl end of OsWRKY62.1 matched with the canonical NES consensus sequence (Φ-X_2-3_-Φ-X_2-3_-Φ-X-Φ, Φ stands for hydrophobic residues such as L, I, or V) (Kosugi et al. 2008). The substitution of V and I with A residues in the NES-like sequence (35S::W62.1^nes4A^-GFP) completely changed the fluorescence distribution expressed in *N. benthamiana* leaf cells in comparison with 35S::W62.1-GFP (Fig. 5c). The data indicate that the NES of OsWRKY62.1 is an important regulatory motif in OsWRKY62.1 translocation.

## Discussion

Nuclear import of immune regulatory proteins and signal transducers is essential for processing plant defense responses against pathogens (Wirthmueller et al. 2013). NTRs such as importin αs are responsible for importing cargo proteins through interaction with NLS sequences. In this study, we identified a special NLS in OsWRKY62 and OsWRKY76, where the concatenated basic amino acids sequence (RK-X_26_-KKK) in the WDs is different from the one found in the common bipartite NLS that is 10–12 residues apart (Chang et al. 2012). Interaction studies indicate that this NLS motif mediates binding with OsIMΔIBBα1a and OsIMΔIBBα1b for nuclear import of OsWRKY62 related proteins (Figs. 2, 3). Similarly, OsIMα1a was found to interact with the AvrPib effector, which contains scattered basic amino acids (Additional file 1: Figure S4). A major difference between the W62WD and AvrPib NLS is that the AvrPib NLS is located in disordered regions, while the W62WD NLS is predicted to be in the β-sheets (Zhang et al. 2018; Additional file 1: Figure S4). The results suggest that the spatial distance between the bipartite NLS is the determinant for interaction with importin α1 NLS binding sites and the number of the concatenated basic amino acids may be responsible for the binding affinity.

OsWRKY62.1-GFP is located exclusively in the cytosol, even in the presence of an additional NLS (W62.1-dsRED^NLS^), implying that other structural features or post-translational modifications affect the OsWRKY62.1 localization. We confirmed that a functional NES at the carboxyl end of OsWRKY62.1 is a determinant factor for its aggregation in cytoplasm (Fig. 5). CRM1/XPO1 recognizes NES motifs of broadly functioning cargo proteins for nuclear export (Fu et al. 2018). Therefore, OsWRKY62 localization is a consequence of competition binding between importins and exportins. OsWRKY62 and OsWRKY76 are proposed to be duplicated genes with very conserved WDs (Wu et al. 2005). However, OsWRKY76.1 has evolved to contain two more consensus NLS motifs other than a similar NLS newly resolved in W62WD. Instead, OsWRKY62.1 has gained an NES motif. OsWRKY62.1 and OsWRKY76.1 form a complex in the nucleus and function similar in repression of disease resistance (Liu et al. 2016). The different localization of OsWRKY62.1 and OsWRKY76.1 may be fine-tuned to avoid excess regulation by this TF couple in disease resistance as well as in response to abiotic stress.

Importin αs may translocate many cargo proteins into nuclei, while the specificity for each importin α is still unclear. Analysis of *OsIMα1a* and *OsIMα1b* overexpression and knockout plants indicated that both OsIMα1a and OsIMα1b function positively in rice disease resistance. Overexpression of *OsIMα1a* or *OsIMα1b* enhanced resistance against *M. oryzae* and increased expression of defense-related genes (Fig. 4). Conversely, *OsIMα1a* and *OsIMα1b* knockout or the double knockout *imα1abKO* mutants showed increased susceptibility to fungal pathogen *M. oryzae*. However, the double *imα1abKO* mutant was not more susceptible than the single gene knockout mutant. A speculation is that translocation of proteins via OsIMα1a or OsIMα1b is a speed limited step. It is supported by the observation that OsWRKY62.1-GFP was predominantly localized in the nuclei when OsIMα1a or OsIMα1b was co-expressed (Fig. 2a, b). Otherwise, the chimeric OsWRKY62.1 was in the cytoplasm even in the presence of endogenous importins (Fig. 2a; Additional file 1: Figure S8). It is also possible that OsIMα1a and OsIMα1b may have partially overlapping functions as observed in importin α3/MOS6 (Palma et al. 2005). The plants overexpressing both *OsIMα1a* and *OsWRKY62.1-GFP* were slightly more susceptible to *M. oryzae* than *OsWRKY62.1-GFP* alone, implying that increased nuclear localization of OsWRKY62.1-GFP attenuates defensive responses. In agreement, overexpression of *OsWRKY62.1* with an additional NES (35S::W62.1-dsRED^NES^) compromised the disease susceptibility caused by OsWRKY62.1 (Fig. 5a, b).

Plant NTRs can be used by pathogens to deliver effector proteins into the host nuclei and manipulate defense responses. Effector HaRxL106 from oomycete pathogen *Hyaloperonospora arabidopsidis* interacts with importin α3/MOS6 for nuclear localization (Wirthmueller et al. 2015). Importin αs are required for translocating effector PvAVH53 of the grapevine oomycete pathogen *Plasmopara viticola* into the nuclei of *N. benthamiana* cells and triggering cell death (Chen et al. 2021). Silencing importin αs expression in *N. benthamiana* or *Vitis vinifera* leads to increased susceptibility to pathogens (Chen et al. 2021), suggesting a positive role of the importin αs against the pathogens tested. Importin αs also mediate transportation of bacterial TALEs to the host nucleus for regulatory functions (Hui et al. 2019; Szurek et al. 2001). Nuclear import of *Xoo* or *Xoc* TALEs increases rice susceptibility to the pathogen, and silencing *OsIMα1a* and *OsIMα1b* enhances resistance to *Xoc* and suppresses the expression of rice susceptibility gene (Hui et al. 2019). Since importin αs have broad cargo targets, the level of host resistance depends on the consequence of the proteins imported in a particular circumstance. In the *OsIMα1a* overexpressing plants, interaction of AvrPib with *OsIMα1a* potentially benefits its nuclear translocation, even though the AvrPib peptide can passively diffuse to the host nucleus if it is secreted from *M. oryzae*. The increased resistance to *M. oryzae* SZ in *OsIMα1a* overexpressing plants (without the corresponding resistance *Pib* gene in ZH17 background) suggests that the elevated defenses through increased *OsIMα1a* expression is dominant over the effects brought by the nuclear localization of AvrPib of SZ strain. Nuclear localization of AvrPib is essential for its avirulence function (Zhang et al. 2018), therefore, how and where AvrPib interacts and whether it interacts directly or indirectly with Pib is an interesting project to be studied. Collectively, we identified a specific NLS and NES in OsWRKY62.1, in which the NES motif plays a dominant role in OsWRKY62.1 localization. Interactions of importin α1s with the specific NLS in the W62WD and the scattered positive-charged amino acids in the AvrPib effector provided a new insight into the NTRs binding with the cargo proteins.

## Conclusion

In the present study, we identified new types of nuclear localization signals in the WRKY domain of OsWRKY62 and AvrPib effector of rice blast fungus, which interact with rice importin OsIMα1a and OsIMα1b for nuclear translocation. OsWRKY62 is generally in the cytosol unless OsIMαs or other nuclear interacting components increase. Nuclear localization of OsWRKY62 is balanced between OsIMαs and XPO1 through association with the nuclear localization signal and nuclear export signal in OsWRKY62, respectively. The AvrPib effector of the rice blast fungus can also use IMα for nuclear translocation. Negative regulators OsWRKY62, OsWRKY76 and the AvrPib effector repress host defense responses.

## Materials and Methods

### Vector Construction and Rice Transformation

The coding sequences of *OsIMα1a* and *OsIMα1b* were amplified from ZH17 cDNA using the gene specific primers listed in Additional file 1: Table S1 along with other primers used in this study. *OsIMα1a* or *OsIMα1b* was fused with C-terminal 3 × myc tag and put under the control of a maize ubiquitin gene promoter to generate overexpression construct (CDU::OsIMα1a or CDU::OsIMα1b). The vectors of 35S::W62.1-GFP, 35S::W62.1-dsRED^NLS^, and 35S::W62.1-dsRED^NES^ were constructed by fusing OsWRKY62.1 respectively with GFP, dsRED^NLS^, and dsRED^NES^, where NLS (SRKEKRMRKV) or NES (NELALKLAGLDINK) was added at the C-terminal end of dsRED, All vectors were modified from binary vector pCambia1300. For CRISPR/Cas9 editing genes, the target sequence of each gene was put under the control of a rice U3 promoter in pOsCas9 vector (Miao et al. 2013). The transgenic plants were obtained from immature rice seeds (*Oryza sativa* L. Zhonghua 17; ZH17) by the *Agrobacterium*-mediated transformation method described previously (Liu et al. 2016). Transgenic plants were verified by PCR amplification and sequencing if required, and the homozygous plants were used in the experiments.

### Plant Growth and Treatments

The seeds of wild-type ZH17 and transgenic plants of T_2_ or higher progeny were surface sterilized and germinated in 1/2 Murashige and Skoog (MS) medium. The seedlings were transferred to 96-hole plates and cultured in 1/4 MS liquid medium at 28 °C with a 16-h/8-h light/dark photoperiod. Leaves of seedlings were treated with 1 µM flg22, or 200 µg/mL chitin in 5 mM MES (4-morpholineethanesulfonic acid, pH 5.8) buffer and sampled at designated time for RNA isolation.

### Pathogen Inoculation

Overexpressing plants of T_2_ or higher progeny were selected on 1/2 MS medium containing 50 mg/L hygromycin for 5 days, transplanted to soil, and grown at 28 °C with a 16-h/8-h light/dark photoperiod. Seeds of the knockout and wild-type ZH17 were germinated without antibiotic selection. Spores were harvested from the PDA medium and suspended in 0.005% Silwet L-77 to 5 × 10^5^ conidia per milliliter. Three-week-old rice plants at seedling stage were inoculated with *M. oryzae* SZ by spraying the spore suspension as described by Liu et al. (2016). Disease severity was estimated by measuring lesion areas 6 days after the infection.

For injection inoculation, the rice plants at tillering stage were injected with spore suspension of *M. oryzae* SZ (5 × 10^4^ spores/mL containing 0.005% Silwet L-77) into the sheath base as described by Liang et al. (2022). The newly grown leaves with disease symptoms were evaluated about 10 days after the inoculation.

### Yeast Two-Hybrid Analysis

The DNA fragments of OsWRKY62, OsWRKY76, OsIMα1a, OsIMα1b, and their mutants were inserted into the pGBKT7 plasmid (Clontech) to generate bait vectors and/or into pGADT7 plasmid (Clontech) to generate prey vectors. Appropriate combinations of bait and prey constructs were transformed into yeast cells and selected on the synthetic dropout (SD) medium lacking Leu and Trp or Leu, Trp, His, and Ade, at 30 °C for 3 days.

### Protein Expression and Pull-Down Analysis

The RK and KKK in the WD of OsWRKY62 (W62WD) were substituted by A residues to generate W62WD^AA^ (mutation RK), W62WD^AAA^ (mutation KKK), and W62WD^5A^ (mutation of both sites), by site-directed mutagenesis. The corresponding cDNAs of *OsIMαΔIBB1a*, *OsIMαΔIBB1b*, *OsWRKY62*, *OsWRKY76*, and their mutants were inserted respectively into a modified pGEX vector (pGEX-tag: 3 × myc or 3 × flag available at the C-terminus). The recombinant proteins were expressed in *Escherichia coli* BL21 (DE3) and purified using Glutathione Sepharose 4B gel (GE Healthcare).

For pull-down assays, proper combinations of the recombinant proteins (1 µg each) were incubated with anti-Flag M2 affinity gel (Sigma) in IP buffer (50 mM Tris–HCl pH 7.4, 150 mM NaCl, 1 mM EDTA, 0.5% Triton X-100, and 0.5% protease inhibitor cocktail (Sigma)) for 3 h at 4 °C. The beads were washed five times with the IP buffer (50 mM Tris–HCl pH 7.4, 150 mM NaCl, 1 mM EDTA, and 0.1% Triton X-100) and then resuspended in 2 × SDS-PAGE loading buffer. The immunocomplexes were separated on 10% polyacrylamide gels and probed with anti-Flag and anti-Myc antibodies (Sigma).

### BiFC and Fluorescence Assays

OsWRKY62.1, OsWRKY76.1, OsIMα1a, OsIMα1b, OsIMβ1, W62WD, and W62WD^5A^ were constructed to fuse in frame with the N-terminal YFP (YFP^N^) or C-terminal YFP (YFP^C^) for BiFC assays. *AvrPib* fragment was amplified from *M. oryzae* SZ cDNA. AvrPib^5A^, mutated at ^28^KK, R^50^, K^52^, and K^70^ to A residues at all sites, was kindly provided by Dr. Yang (Zhang et al. 2018). AvrPib^7A^ was obtained by further substitution of K^41^ and R^45^ in AvrPib^5A^ to A residues, by site-directed mutagenesis. AvrPib and its mutants were fused with YFP^N^. Also, W62WD and W62WD^5A^ were cloned to fuse with the chimeric GFP-GUS protein, and OsIMα, OsIMαΔIBB1a, and OsIMαΔIBB1b were fused with dsRED, respectively. All the chimeric genes were put under the control of 35S promoter and the obtained plasmids were introduced into *Agrobacterium tumefaciens* EHA105 strain. Agrobacteria containing appropriate plasmids were co-infiltrated into leaves of four-week-old *N. benthamiana* plants, and kept in a growth-chamber at 25 °C with a 16 h -light/8 h dark photoperiod for 2–3 days. Fluorescence images were analyzed on a laser scanning confocal microscope (SP8, Leica) with the following excitation and emission wavelengths: GFP and YFP (Ex: 488 nm, Em: 505–550 nm), RED (Ex: 552 nm, Em: 570–610 nm), and DAPI (Ex: 360 nm, Em: 430–480 nm).

### Quantitative Reverse Transcription PCR (qRT-PCR) Analysis

Total RNAs of various tissues were extracted using the Trizol method and treated with DNase I to remove possible DNA contaminations. The first strand cDNA was synthesized using a reverse transcription kit (TaKaRa). The relative transcript levels were quantified using TB Green Premix Ex Taq (TaKaRa). Two biological replicates were performed, and the relative gene expression was calculated by 2^−^^ΔΔ^^Ct^. The rice *ubiquitin* gene was used as an internal control. Gene-specific primers used in qRT-PCR were listed in Additional file 1: Table S1.

### Structure Simulation

The structure of W62WD was simulated based on AtWRKY1WD (PDB code: 2AYD) using homology-modeling by SWISS-MODEL server (https://swissmodel.expasy.org). Specific steps refer to Waterhouse's method (Waterhouse et al. 2018).

## Supplementary Information

**Additional file 1.Fig. S1. Analysis of OsWRKY62 and OsWRKY76 interacting with OsIMα1 in yeast. (A)** Analysis of OsWRKY76 (W76.1) and its deletion mutants, and OsIM**α**∆IBB1 auto-activation. **(B)** Schematic diagrams of OsWRKY62.1 (W62.1) and its deletion mutants. **(C)** Analysis of OsWRKY62 and its mutants interacting with OsIM**α**∆IBB1. Yeast cells with serial dilutions were incubated in synthetic dropout medium lacking Leu and Trp (left) or Leu, Trp, His, and Ade (right) and photographed 3 d after plating. Yeast cells harboring AD-T with BD-53 or BD-Lam vectors were used as the positive or negative control, respectively. **Fig. S2. Phylogenetic analysis of importin αs.** Importin αs from *Oryza sativa* (Os), *Lycopersicon esculentum* (Le), and *Arabidopsis thaliana* (At) were compared. Multiple sequence alignments of amino acid sequences were generated using ClustalW in MEGA7.0. The sequence alignments obtained were used as input for the neighbor-joining method using MEGA7.0 to construct the phylogenetic tree. For phylogenetic tree construction, a bootstrap method with 1,000 replications was used for test of phylogeny. Scale bar indicates 0.2 amino acid substitution per site. **Fig. S3. OsIM**Δ**IBBα1a interacting with OsIMβ1 and increased OsWRKY62.1-GFP nuclear localization through overexpressing OsIMα1a.** (**A**) BiFC visualizations of IMα1a and IMα∆IBB1a interacting with IMβ1. IMβ1 was fused in frame with YFP N-terminal region (YFP^N^) and IMα1a and IMα∆IBB1a were fused with YFP C-terminal region (YFP^C^). The plasmids indicated were introduced into *N. benthamiana* leaves through agroinfiltration method. Red fluorescence (dsRED^NLS^) shows nuclear localization. From left panels to right: YFP images (YFP), dsRED images (RED), and combined YFP and RED in the bright field (Merged). (**B**) Sheaths from three-week-old 35S::OsWRKY62.1-GFP (35S:: W62.1-GFP) and 35S::OsWRKY62.1-GFP/CDU::IMα1a (genetic cross progeny) plants were used. DAPI for nuclear staining. From top panels to bottom: DAPI, GFP, DIC, and the bright field image combined the fluorescent images (Merged). **Fig. S4. Simulated structures of AvrPib and the WRKY domain of OsWRKY62.1. (A)** The structure of AvrPib was from Zhang et al. (2018). The positive-charged amino acids of AvrPib are shown in red in the structure. **(B)** The structure of W62WD is simulated based on AtWRKY1WD (PDB code: 2AYD) using homology-modeling by SWISS-MODEL server. **Fig. S5. Information of OsIMα1 knockout mutants.** (**A**) Knockout mutant of *OsIMα1a* (*imα1aKO*). (**B**) Knockout mutant of *OsIMα1b* (*imα1bKO*). The sequences of the target sites are shown in green and the inserted nucleotides are in blue. **Fig. S6. OsIMα1 positively regulated resistance against rice blast fungus.** (**A**) Three-week-old transgenic and wild-type (ZH17) plants were inoculated with *M. oryzae* SZ (5 × 10^5^ spores/mL) by foliar spraying. Photographs were taken six days after the inoculation. Bar = 2 cm. (**B**) *P*-values were calculated by one-tailed Student's *t*-test. Prefix CDU for *OsIMα1a* and *OsIMα1b* overexpressing plants and suffix KO for the knockout lines. **Fig. S7. Induction of OsIMα1 expression. (A)** Induction of *OsIMα1a* and *OsIMα1b* expression by flg22 (1 µM) or chitin (200 µg/mL) treatment. (**B**) Induction of *OsIMα1a* and *OsIMα1b* expression by *M. oryzae* SZ. Transcriptional levels of *OsWRKY62* (**C**) and *OsWRKY76* (**D**) in their overexpression plants. (**E**) Expression of *OsIMα1a* and *OsIMα1b* in *OsWRKY62* and *OsWRKY76* overexpressing and knockout plants. **Fig. S8. Analysis of OsWRKY62.1 localization.** Sheaths of 35S::W62.1-dsRED^NLS^ and 35S::W62.1-dsRED^NES^ rice plants were used for fluorescence observation. DAPI for nucleus staining. From top panels to bottom: DAPI, RED, DIC, and the bright field image combined the fluorescent images (Merged). Bar = 20 µm. **Table S1** Primers used in this study.

## Data Availability

All data supporting the findings of this study are available within the paper and within its supplementary materials published online.

## Abbreviations

BiFC: Bi-molecular fluorescence complementation
EDS1: Enhanced disease susceptibility 1
IBB: Importin β-binding
*M. oryzae*: *Magnaporthe oryzae*
*mos*: *Modifiers of snc1*
MPK: Mitogen-activated protein kinase
NES: Nuclear export signal
NLS: Nuclear localization signal
NPCs: Nuclear pore complexes
NPR1: Nonexpresser of pathogenesis-related gene 1
NTRs: Nuclear transport receptors
*snc1*: *Suppressor of npr1-1, constitutive1*
TALEs: Transcription activator-like effectors
TF: Transcription factor
WD: WRKY domain
*Xoo*: *Xanthomonas oryzae* pv. *oryzae*
*Xoc*: *Xanthomonas oryzae* pv. *oryzicola*
