# Supplementary material for: OsWRKY62 and OsWRKY76 Interact with Importin α1s for Negative Regulation of Defensive Responses in Rice Nucleus
Source: Rice (N Y). 2022 Feb 20;15:12. doi: 10.1186/s12284-022-00558-4 (PMC8859016; doi:10.1186/s12284-022-00558-4)
Supplement: Supplementary file 1 — Additional file 1.Fig. S1. Analysis of OsWRKY62 and OsWRKY76 interacting with OsIMα1 in yeast. (A) Analysis of OsWRKY76 (W76.1) and its deletion mutants, and OsIMα∆IBB1 auto-activation. (B) Schematic diagrams of OsWRKY62.1 (W62.1) and its deletion mutants. (C) Analysis of OsWRKY62 and its mutants interacting with OsIMα∆IBB1. Yeast cells with serial dilutions were incubated in synthetic dropout medium lacking Leu and Trp (left) or Leu, Trp, His, and Ade (right) and photographed 3 d after plating. Yeast cells harboring AD-T with BD-53 or BD-Lam vectors were used as the positive or negative control, respectively. Fig. S2. Phylogenetic analysis of importin αs. Importin αs from Oryza sativa (Os), Lycopersicon esculentum (Le), and Arabidopsis thaliana (At) were compared. Multiple sequence alignments of amino acid sequences were generated using ClustalW in MEGA7.0. The sequence alignments obtained were used as input for the neighbor-joining method using MEGA7.0 to construct the phylogenetic tree. For phylogenetic tree construction, a bootstrap method with 1,000 replications was used for test of phylogeny. Scale bar indicates 0.2 amino acid substitution per site. Fig. S3. OsIMΔIBBα1a interacting with OsIMβ1 and increased OsWRKY62.1-GFP nuclear localization through overexpressing OsIMα1a. (A) BiFC visualizations of IMα1a and IMα∆IBB1a interacting with IMβ1. IMβ1 was fused in frame with YFP N-terminal region (YFPN) and IMα1a and IMα∆IBB1a were fused with YFP C-terminal region (YFPC). The plasmids indicated were introduced into N. benthamiana leaves through agroinfiltration method. Red fluorescence (dsREDNLS) shows nuclear localization. From left panels to right: YFP images (YFP), dsRED images (RED), and combined YFP and RED in the bright field (Merged). (B) Sheaths from three-week-old 35S::OsWRKY62.1-GFP (35S:: W62.1-GFP) and 35S::OsWRKY62.1-GFP/CDU::IMα1a (genetic cross progeny) plants were used. DAPI for nuclear staining. From top panels to bottom: DAPI, GFP, DIC, and the b [file 12284_2022_558_MOESM1_ESM.pdf]

Supplementary Figure S1

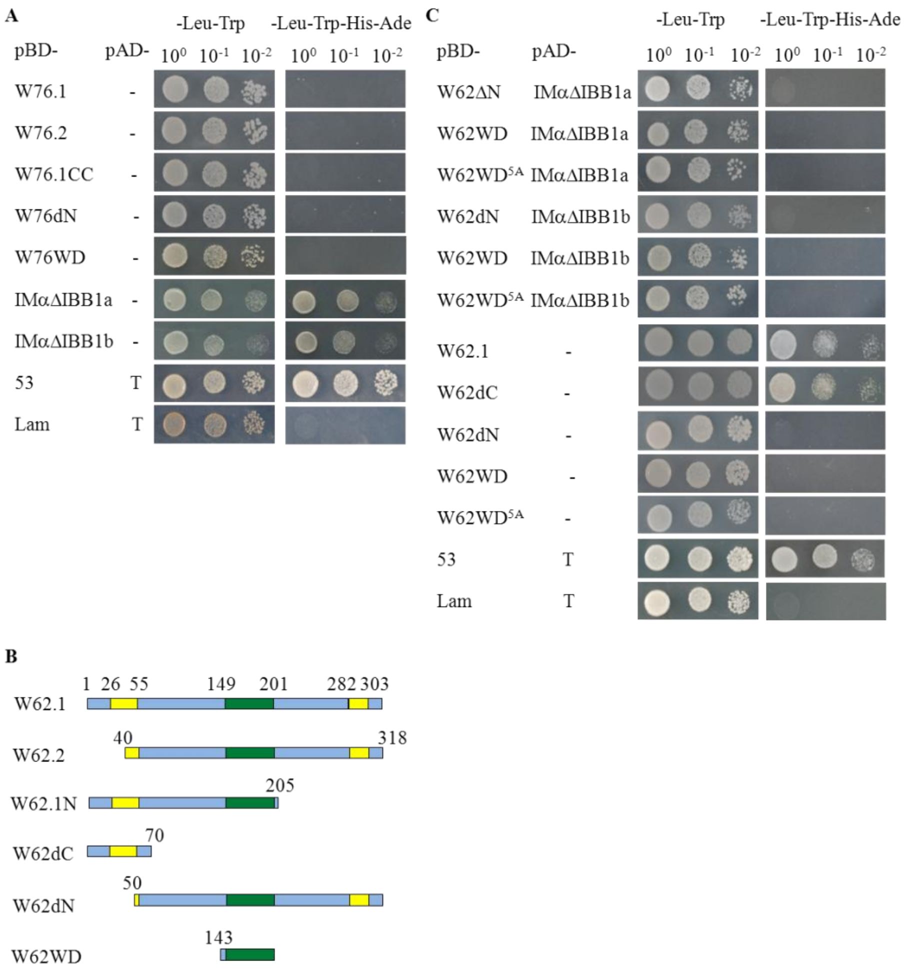

Supplementary Figure S1. Analysis of OsWRKY62 and OsWRKY76 interacting with OsIMα1 in yeast.

(A) Analysis of OsWRKY76 (W76.1) and its deletion mutants, and OsIMαΔIBB1 auto-activation. **(B)** Schematic diagrams of OsWRKY62.1 (W62.1) and its deletion mutants. **(C)** Analysis of OsWRKY62 and its mutants interacting with OsIMαΔIBB1. Yeast cells with serial dilutions were incubated in synthetic dropout medium lacking Leu and Trp (left) or Leu, Trp, His, and Ade (right) and photographed 3 d after plating. Yeast cells harboring AD-T with BD-53 or BD-Lam vectors were used as the positive or negative control, respectively.

Supplementary Figure S2

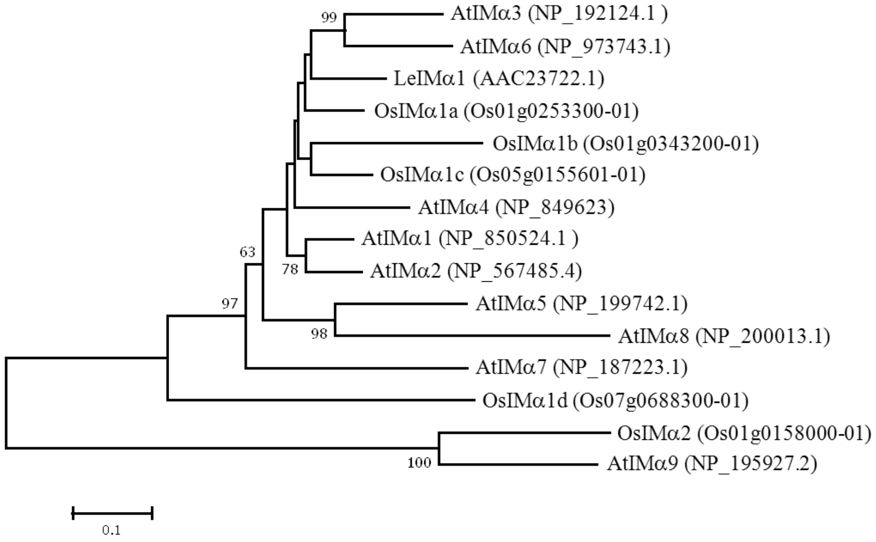

Supplementary Figure S2. Phylogenetic analysis of importin αs.

Importin αs from *Oryza sativa* (Os), *Lycopersicon esculentum* (Le), and *Arabidopsis thaliana* (At) were compared. Multiple sequence alignments of amino acid sequences were generated using ClustalW in MEGA7.0. The sequence alignments obtained were used as input for the neighbor-joining method using MEGA7.0 to construct the phylogenetic tree. For phylogenetic tree construction, a bootstrap method with 1,000 replications was used for test of phylogeny. Scale bar indicates 0.1 amino acid substitution per site.

Supplementary Figure S3

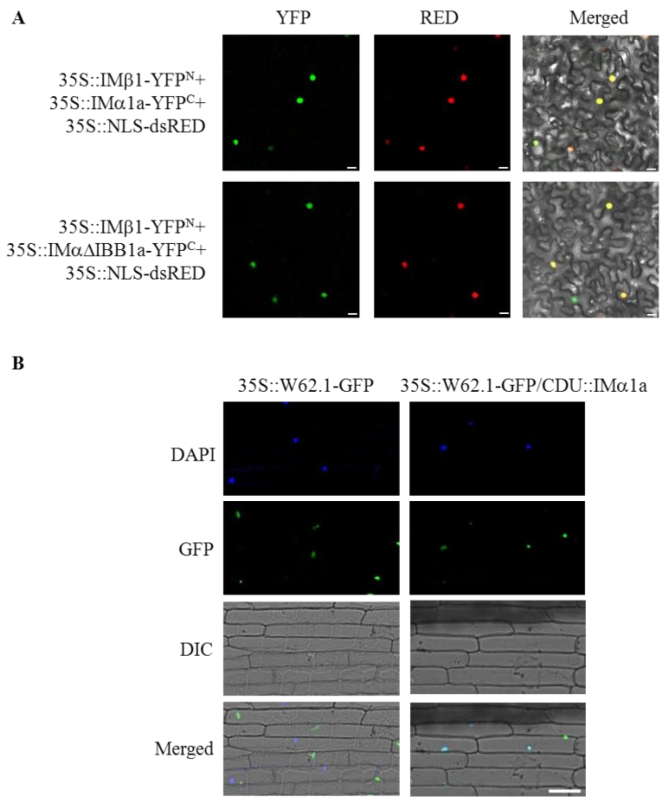

Supplementary Figure S3. OsIMΔIBBα1a interacting with OsIMβ1 and increased OsWRKY62.1-GFP nuclear localization through overexpressing OsIMα1a.

(**A**) BiFC visualizations of IMα1a and IMαΔIBB1a interacting with IMβ1. IMβ1 was fused in frame with YFP N-terminal region (YFP^N) and IMα1a and IMαΔIBB1a were fused with YFP C-terminal region (YFP^C). The plasmids indicated were introduced into *N. benthamiana* leaves through agroinfiltration method. Red fluorescence ($dsRED^{NLS}$) shows nuclear localization. From left panels to right: YFP images (YFP), dsRED images (RED), and combined YFP and RED in the bright field (Merged). (**B**) Sheaths from three-week-old 35S::OsWRKY62.1-GFP (35S:: W62.1-GFP) and 35S::OsWRKY62.1-GFP/CDU::IMα1a (genetic cross progeny) plants were used. DAPI for nuclear staining. From top panels to bottom: DAPI, GFP, DIC, and the bright field image combined the fluorescent images (Merged).

Supplementary Figure S4

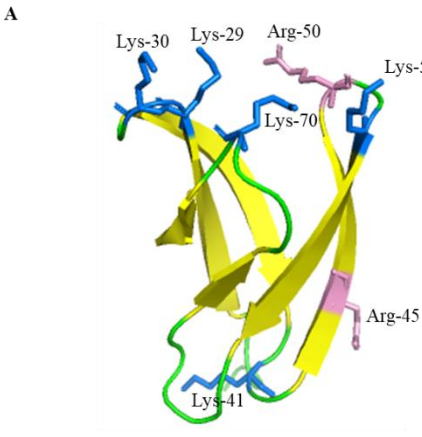

AvrPib sequence

1 10 20
MRSSTTFITIAFIAMQALGIAA
30 40 50 60 70
TQVTILKKGERITWVEVPKGESREFNIRGKYFTVSVSDDGTPSISGSKYTVE

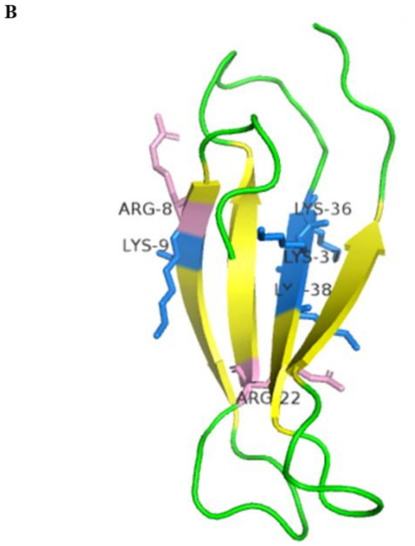

Supplementary Figure S4. Simulated structures of AvrPib and the WRKY domain of OsWRKY62.1.

(A) The structure of AvrPib was from Zhang et al. (2018). The positive-charged amino acids of AvrPib are shown in red in the structure. **(B)** The structure of W62WD is simulated based on AtWRKY1WD (PDB code: 2AYD) using homology-modeling by SWISS-MODEL server.

Supplementary Figure S5

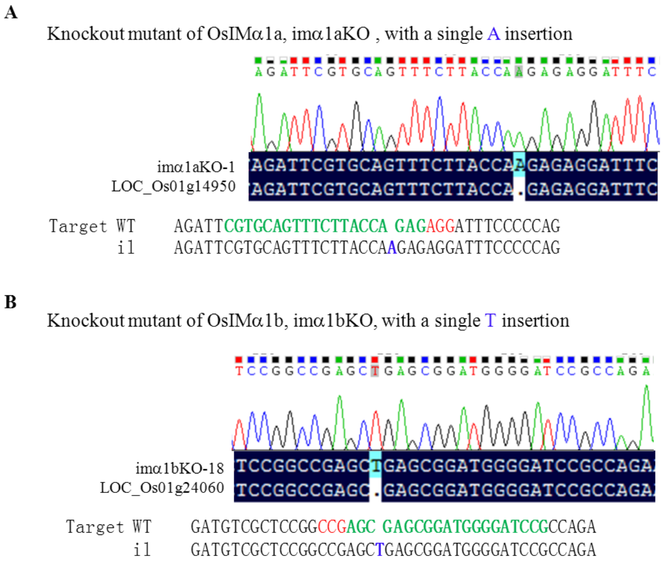

Supplementary Figure S5. Information of *OsIMα1* knockout mutants.

(**A**) Knockout mutant of *OsIMα1a* (*imα1aKO*). (**B**) Knockout mutant of *OsIMα1b* (*imα1bKO*). The sequences of the target sites are shown in green and the inserted nucleotides are in blue.

Supplementary Figure S6

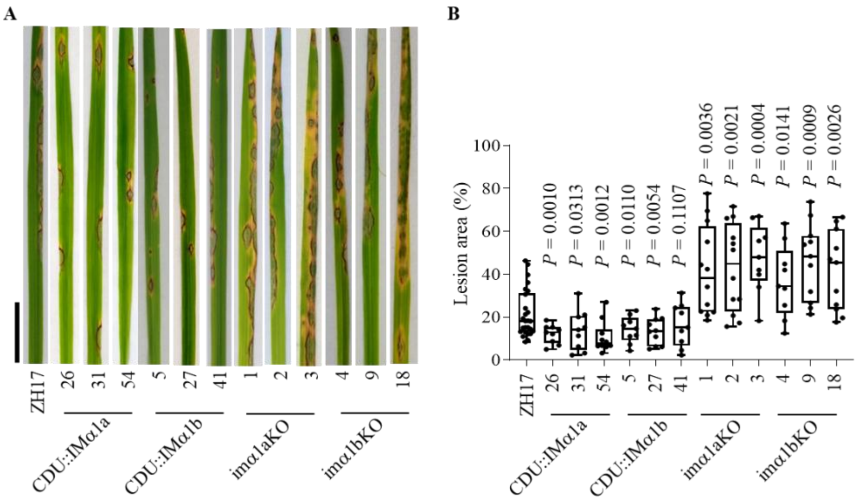

Supplementary Figure S6. OsIMα1 positively regulated resistance against rice blast fungus. (**A**) Three-week-old transgenic and wild-type (ZH17) plants were inoculated with *M. oryzae* SZ (5×10^5 spores/mL) by foliar spraying. Photographs were taken six days after the inoculation. Bar = 2 cm. (**B**) *P*-values were calculated by one-tailed Student's *t*-test. Prefix CDU for *OsIMα1a* and *OsIMα1b* overexpressing plants and suffix KO for the knockout lines.

Supplementary Figure S7

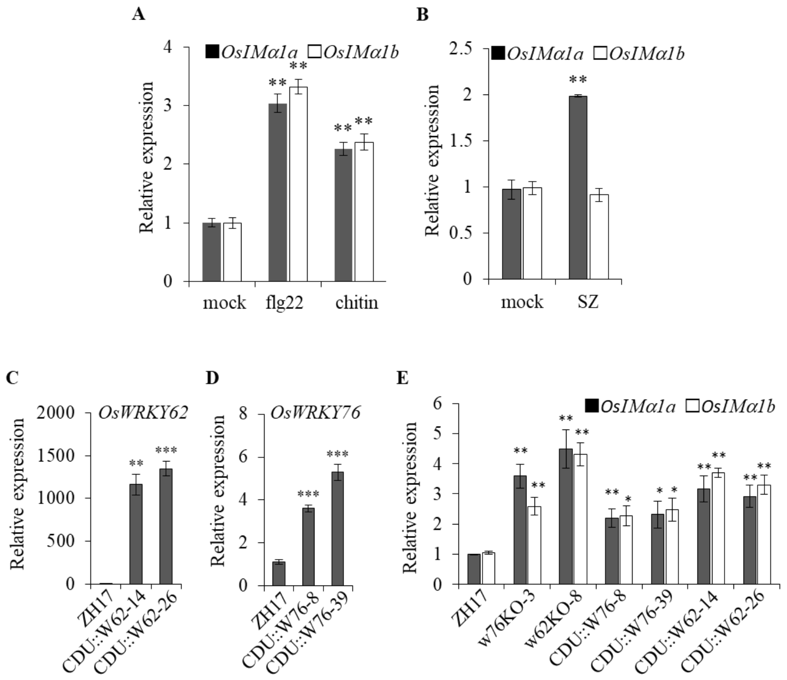

Supplementary Figure S7. Induction of *OsIMα1* expression.

(A) Induction of *OsIMα1a* and *OsIMα1b* expression by flg22 (1 μM) or chitin (200 μg/mL) treatment. (**B**) Induction of *OsIMα1a* and *OsIMα1b* expression by *M. oryzae* SZ. Transcriptional levels of *OsWRKY62* (**C**) and *OsWRKY76* (**D**) in their overexpression plants. (**E**) Expression of *OsIMα1a* and *OsIMα1b* in *OsWRKY62* and *OsWRKY76* overexpressing and knockout plants.

Supplementary Figure S8

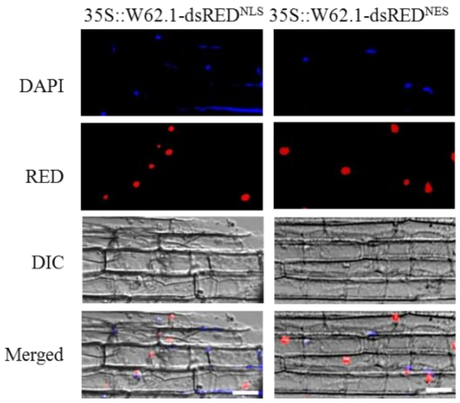

Supplementary Figure S8. Analysis of OsWRKY62.1 localization.
Sheaths of 35S::W62.1-dsRED^{NLS} and 35S::W62.1-dsRED^{NES} rice plants were used for fluorescence observation. DAPI for nucleus staining. From top panels to bottom: DAPI, RED, DIC, and the bright field image combined the fluorescent images (Merged). Bar = 20 μm.

Supplementary Table S1 Primers used in this study

Supplement Table S1 Primers used in this study

Primer	gene ID	Forward sequence	Reverse sequence
For qRT-PCR			
qOsWRKY62.1	LOC_Os09g25070.1	5'-TTCAGCCGATCGCCGGCCGAG-3'	5'-GCGTGGTGACCGGCCAGAAT -3'
qOsWRKY76	LOC_Os09g25060	5'-AGGTCGCGTCGCCGGAGTTC-3'	5'-TCGGGCAGCTTCTGGAGGATCG-3'
qOsPR1a	LOC_Os07g03710	5'-GAGCTCGTGCGGGCACTACAC-3'	5'-TGATGAAGACGCCGAGGTCGC-3
qOsPR1b	LOC_Os01g28450	5'-ATCTATGTAGCCGGATTGTGTG-3'	5'-CACAGCGACGTCGTTTATTCC-3'
qOsLOX2	LOC_Os03g08220	5'-CTGCCGTACCAGCTGATGAAGC-3'	5'-AGATTTGGGAGTGACATATTGGTT-3'
qOsWRKY45	LOC_Os05g25770	5'-AATCGTCCGGGAATTCGGTG-3'	5'-GAAGTAGGCCTTTGGGTGCT-3'
qOsIMα1a	LOC_Os01g14950	5’-AGATGACGCGCAAACTCAGTGC-3’	5’-GTCAACAGACTTAGGAGGCAAGGG-3’
qOsIMα1b	LOC_Os01g24060	5’-TTGCATCAAGCCGCTTTGTG-3’	5’-ATCAAGGGCCTCCAAACAAG-3’
qOsUbiquitin	LOC_Os05g06770	5'-GTGGTGGCCAGTAAGTCCTC-3'	5'-GGACACAATGATTAGGGATCA-3'
For site-directed mutagenesis			
WRKY62WD^{2A}	LOC_Os09g25070.1	5’-GATGGGTACCAATGGGCGGCGTACGGGCAGAAGGTG-3’	5’-CACCTTCTGCCCGTACGCCGCCCATTGGTACCCATC-3’
WRKY62WD^{3A}	LOC_Os09g25070.1	5’-CCGTCTTGCCCCGTCGCGGCGGCGCTCCAAAGATGTGCG-3’	5’-CGCACATCTTTGGAGCGCCGCCGCGACGGGGCAAGACGG-3’
W62^{NES}	LOC_Os09g25070.1	5'-GTTGGGGATCAAGGCCCACACGGTCCTGGTCATTTGCACGTG-3’	5’-CACGTGCAAATGACCAGGACCGTGTGGGCCTTGATCCCCAAC-3’
AvrPib^{7A}	KM887844.1	5’-GTGGAAGTGCCGGCTGGCGAATCTGCTGAATTTAATATT-3’	5'-AATATTAAATTCAGCAGATTCGCCAGCCGGCACTTCCAC-3'
For protein expression and yeast assay			
OsIMα1a	LOC_Os01g14950	5’-AGGATCCGAGCCAGCCATGTCGCTGC-3'	5’-TGGTCGACTTTGAATTGAGCAGCACCACCG-3'
OsIMα1b	LOC_Os01g24060	5’-TACATATGTCGGCGATGTCGCTCC-3'	5’-ACCTCGAGCTACGGTGCATTTCCATCCA-3'
OsIMαΔIBB1a	LOC_Os01g14950	5'-TTGGATCCGCCATGATTGGTGGAGTTTATTCG-3'	5'-TGGTCGACTTTGAATTGAGCAGCACCACCG-3'
OsIMαΔIBB1b	LOC_Os01g24060	5'-TTGGATCCGCGATGATGGTGCAGGGGTTGT-3'	5'-ACCTCGAGCTACGGTGCATTTCCATCCA-3'
OsIMβ1	LOC_Os05g28510	5'-TTAGATCTCACGCCATGAATATCACTCAAATC-3'	5'-GCGTCGACCCCGGGAGAAACCAGTGCTTGGTTTATCA-3'
OsWRKY62ΔN	LOC_Os09g25070.1	:5'-AGGGATCCCGAATGCTCGACGCCATTCTGG-3'	5'-AAGCTCGAGCAAATGAACAGGAATGTGTGGGAT-3'

OsWRKY62ΔC	LOC_Os09g25070.1	5'-AGTCCCGGGCTTAGCTGCCGCCATGGACGAC-3'	5'-ATCTCGAGCGACAGCGACGGCGCCAAGAG-3'
OsWRKY62.1	LOC_Os09g25070.1	5'-AGTGGATCCCTTAGCTGCCGCCATGGACGAC-3'	5'-AAGAAGCTTCCCGGGCAA ATGAACAGGAATGTGTGGGAT-3'
OsWRKY62.2	LOC_Os09g25070.2	5'-AGTGGATCCATGGAGGAGAACGCGCGG-3'	5'-AAGAAGCTTCCCGGGCAAATGAACAGGAATGTGTGGGAT-3'
OsWRKY62WD	LOC_Os09g25070.1	5'-AAGGATCCGCCATGGACGTGAAGGATGGGTACCAATGG-3'	5'-GTCACGTGCGTGGACAGGGCATGGTTGTG-3'
OsWRKY76.1	LOC_Os09g25060.1	5'-AGTGGATCCACTAGTTCGTCGTCGTCGATGGACG-3'	5'-GGCCCGGGGAATTCGGGCAGCTTCT-3'
OsWRKY76.2	LOC_Os09g25060.2	5'-AGTGGATCCACTAGTTCGTCGTCGTCGATGGACG-3'	5'-GGCCCGGGGAATTCGGGCAGCTTCT-3'
OsWRKY76WD	LOC_Os09g25060.1	5'-GTGGATCCGCCATGGACGTGAAG-3'	5'-AGCACGTGCGTGGACAGGGCA-3'
